# Parents' willingness to pay for the prevention of childhood overweight and obesity

**DOI:** 10.1186/s13561-014-0020-8

**Published:** 2014-09-16

**Authors:** Dorothea Kesztyüs, Romy Lauer, Anja C Schreiber, Tibor Kesztyüs, Reinhold Kilian, Jürgen M Steinacker

**Affiliations:** Division of Sports and Rehabilitation Medicine, Department of Internal Medicine II, Ulm University Medical Centre, Frauensteige 6, Haus 58/33, Ulm, D-89075 Germany; Department of Computer Science, University of Applied Sciences, Ulm, Germany; Section Health Economics and Health Services Research, Department of Psychiatry II, Ulm University, Günzburg, Germany

**Keywords:** Child, Overweight, Obesity/prevention & control, Obesity/economics, Germany

## Abstract

**Objective:**

To determine parental willingness-to-pay (WTP) for childhood obesity prevention.

**Methods:**

Cross-sectional data from the follow-up measurements (2011) of a health promotion programme in German primary schools. Data collection included anthropometric measurements of children and self-administered questionnaires for parents, including WTP assessment. Mann-Whitney U-Test was used for differences between groups, and regression analysis to identify factors associated with general WTP and amount of WTP.

**Results:**

From 1 534 parents, 97.8% considered overweight/obesity to be serious public health problems. A general WTP to reduce the incidence of childhood overweight/obesity by half, was declared by 48.8%. Parents of overweight/obese children showed with 61.4%, significantly more frequently, their general WTP than the others with 47.2% (*p* = 0.001). Mean WTP was €23.04 (99% confidence interval (CI) [22.45; 23.75]) per month. Parents of centrally obese children showed significantly higher WTP than parents of the other children (*p* = 0.001). General WTP and the amount of WTP were associated with the central obesity of the child, migration status and household income. Additionally, general WTP was associated with maternal obesity.

**Conclusions:**

Nearly half of the parents were willing to invest in prevention of obesity. The general WTP significantly occurs more often and with higher amount in affected parents.

**Electronic supplementary material:**

The online version of this article (doi:10.1186/s13561-014-0020-8) contains supplementary material, which is available to authorized users.

## Background

The ongoing threat from the worldwide overweight and obesity epidemic is far from being under control. Although there is some evidence that the prevalence of obesity in youth, defined by body mass index (BMI), is plateauing [1], other researchers find central obesity to be rising instead [2]-[4]. Most of the obesity-related health risks are strongly associated with a high waist circumference (WC) [5]. Primary school children with a waist-to-height ratio (WHtR) beyond the boundary value of 0.5 have more visits to a physician and more sick days than their normal weight peers [6]. Altogether, this leads to higher health care costs or a higher utilization of health care services for obese children [7]-[9]. Trasande detected in his cost of illness approach, that the economic consequences of childhood obesity will be more profound than previously documented [10].

A growing number of preventive measures and health promotion programs have been implemented to try to reverse the trend and to help children develop a healthy lifestyle, but only few have been evaluated with regard to their cost-effectiveness [11]. In this respect, one successful prevention programme was URMEL-ICE (Ulm research on Metabolism, Exercise and Lifestyle Intervention in Children, 2006 - 2009 [12]), with a hypothetical threshold for cost-effectiveness of an annual €35 maximum “willingness-to-pay” (WTP) [13]. This WTP covers the prevented increase in waist circumference as well as the prevented increase in WHtR. The respective incremental cost-effectiveness ratios were €11.11 for one centimetre of WC and €18.55 for one unit (0.01) WHtR increase prevented. Furthermore, the URMEL-ICE intervention cut the risk of incident central obesity by half [13].

In a contingent valuation analysis of the WTP to reduce childhood obesity in New York state residents, Cawley observed a mean value of €46.41 ($36.83 in 2006) for a 50% reduction in childhood obesity [14]. Other research on WTP in the field of obesity was identified for secondary/tertiary prevention and therapy [15],[16]. Sikorski et al. showed that obesity prevention support in Germany is high, and a vast majority of participants in their study proclaimed general willingness to pay for their participation in preventive programmes [17]. More discussion concerning the WTP is observed for “quality adjusted life years” (QALYs) [18] whereas the QALY itself may be regarded as a competing construct to the WTP [19], both being measures of the value of reductions in health risks.

The aim of this study is to evaluate the real WTP in a sample of parents of primary school children, taking part in the evaluation of the health promotion programme “Join the Healthy Boat” in southern Germany [20].

## Participants and methods

### Overview of the Baden-Württemberg study

The Baden-Württemberg Study is a randomized, controlled study to evaluate the school-based health promotion programme “Join the Healthy Boat” in primary school children in southern Germany. A detailed description of this study has been published elsewhere [20]. The study protocol was approved by the ethics committee of Ulm University in June 2009 (Application No. 126/10). The Baden-Württemberg Study is registered at the German Clinical Trials Register (DRKS), Freiburg University, Germany, under the DRKS-ID: DRKS00000494.

The “Join the Healthy Boat” intervention was designed by scientists and dedicated teachers with the aim of leading children to adopt a healthy lifestyle through daily lessons and exercise. Health messages and physical activity are integrated in the curriculum and communicated by the teachers during regular lessons at school. For the dissemination of the programme, a “train-the-trainer” model resulting in a peer-to-peer approach for teachers was implemented by the scientists of Ulm University [21]. This approach was assumed to be cost-saving and sustainable in comparison to an expert driven intervention, where specially trained professionals are sent to schools for a couple of lessons over a fixed period.

The study started in 2010 before the beginning of the intervention with a baseline data collection. After one year of intervention in 2011, follow-up data collection was conducted and in the corresponding parental questionnaire, participants were asked about their WTP.

### Participants

Teachers who registered in 2010 for the training to implement the health promotion programme “Join the Healthy Boat” were asked to participate in the outcome evaluation. This resulted in 157 teachers in 86 schools for the baseline measurements and 154 teachers in 84 schools for the one-year follow-up data collection. Parents gave their written informed consent and 1 947 children took part in the first measurements. Data from 1 829 children and 1 593 parental questionnaires were available from the follow-up procedure.

**Figure 1:**
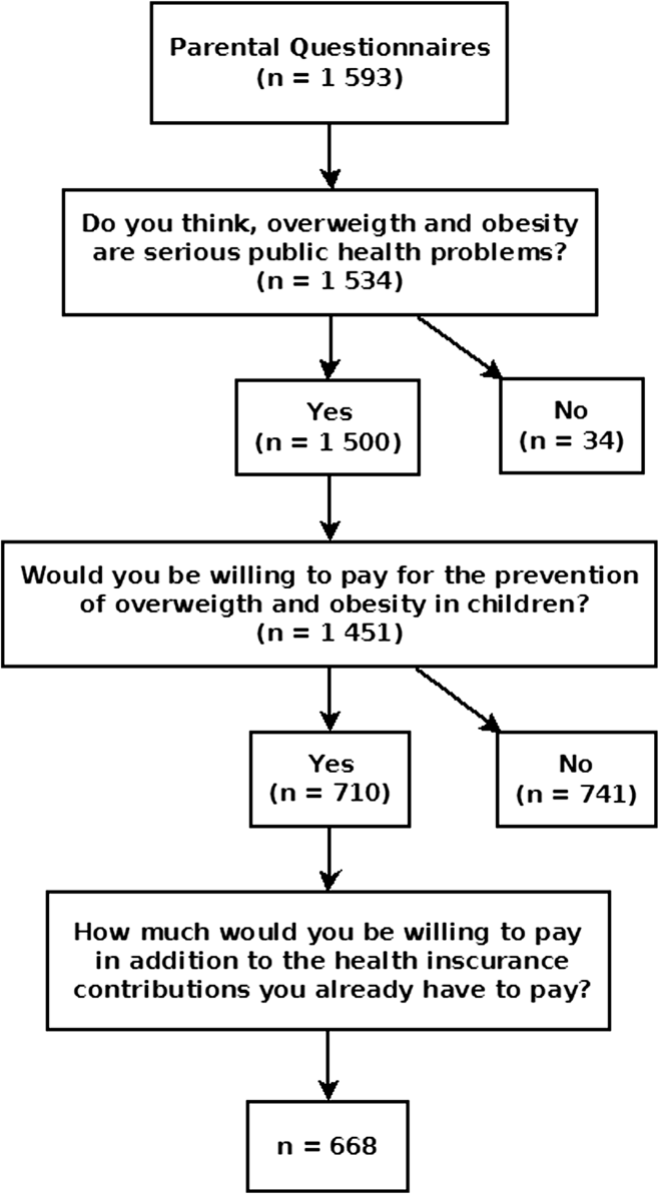
**Overview of datasets available for the analysis of the parental willingness to pay (WTP).** Flow chart showing the respective underlying number of datasets for each part of the analysis.

An overview of the respective underlying number of datasets available for the analysis is shown in the flowchart in Figure 1.

### Data collection

Data collection included anthropometric measurements of the children in a standardized manner and self-administered questionnaires for the parents. The follow-up data collection took part from September to November 2011. All data were checked for their plausibility.

### Questionnaires and derived variables

Parents were asked to fill in questionnaires to gather information about demographics, health and behavioural topics, their attitude towards overweight and obesity and their willingness to pay for the reduction of childhood overweight and obesity.

#### Parental health related variables

Parental body mass index (BMI) was computed as weight (kilogram) divided by height (meter) squared, as self-reported in the questionnaires, and categorised as overweight (BMI???25.0), and obesity (BMI???30.0), according to the international classification of the World Health Organization (WHO) [22]. Self-reported waist circumference divided by height was used to calculate WHtR and was classified as central obesity above or equal to the threshold of 0.5 as recommended by Ashwell and Hsieh [23].

Parents were asked to rate their level of health awareness on a four point rating scale which was then dichotomised into a high and a low-level, putting the upper levels (high, very high) and the lower levels (little, very little) together, respectively. Furthermore, as a more objective measure of a healthy lifestyle the parents were asked whether they were current smokers, non-smokers or ex-smokers.

#### Socio-economic variables

Family education level was ranked in accordance with the CASMIN classification as the highest level of two parents or the level of a single parent [24]. It was dichotomised for analysis into tertiary level, on the one side, versus primary and secondary level on the other side. Monthly household income was grouped into a low (< €2 250) a medium (≥ €2 250 and < €4 000) and a high (≥ €4 000) category.

A migration background of the child was assumed if at least one parent was born abroad or at least one parent mainly spoke a foreign language during the child's first years of life.

#### Willingness to pay and related variables

WTP represents a method to measure an economic value, in health economics the value in question is a change in health status. Contingent valuation (CV) uses the stated preference of the individual to elicit the monetary valuation of health improvements. In CV studies, participants are asked directly (e.g. open-ended questions) or indirectly (e.g. interval checklist) how much they are willing to pay for a certain benefit in health. Several techniques can be used to conduct CV studies; like face-to-face interviews, telephone interviews or mail surveys. Relevant co-variables that are associated with the WTP, have to be identified to adequately adjust the results.

The WTP section of the questionnaire for the present study was started with some information about overweight and obesity with regard to the prevalence, the health risks and the health care costs. Then participants were asked, whether they think that overweight and obesity are serious public health problems. They were requested to imagine a preventive measure that cuts the incidence of childhood overweight and obesity by half, and asked whether they would be willing to pay for this prevention (general WTP). Those who answered with “yes” were additionally asked to state the amount of money they were willing to pay per month (amount of WTP). Answer categories started with “€1 - €5” and went up to “€301 - €500” in 10 steps. The last category was open with “more than €500, namely €□□□□”.

The consideration of the weight status of their own child was considered to be an important background variable for the parental willingness to pay. Therefore parents were asked whether they considered their child to be too corpulent or too thin on a five-point rating scale. The answers were then dichotomised, putting “very thin”, “a bit thin” and “neither/nor” together in one category and “a bit corpulent” and “very corpulent” in the other category. Furthermore, parents were asked whether being thin is important for being attractive. The four point rating scale used for this consideration was dichotomised for analysis into “not important” vs. “important”.

### Anthropometric measurements

Anthropometric measurements were performed by trained staff according to ISAK-standards [25]. The children's BMI was computed as weight (kilogram) divided by height (meter) squared, and percentiles were allocated according to the German reference data from Kromeyer-Hauschild [26]. The 90^th^ and 97^th^ sex and age specific percentiles were used to define overweight and obesity. WHtR was calculated as the ratio of WC and height and central obesity was defined as WHtR ≥ 0.5.

### Statistical analyses

Differences between those parents who were in general willing to pay and those who were not, were analysed using the Mann—Whitney-*U* test for continuous data and the exact Fisher test for categorical data. The significance level was set at α <0.05 for two-sided tests.

The general WTP was analysed using a logistic regression model. As potential explanatory variables, all variables listed in Table 1 were included in the modelling process, based on the relevance of content and significance of association with the outcome. The same applies to the amount of WTP which was analysed in a bootstrap interval regression model for those participants who confirmed their WTP by choosing the corresponding values in the questionnaire, but excluding those who were not willing to pay in general. Based on the maximum likelihood function, the interval regression estimates the probability that a latent variable exceeds one threshold but is less than another threshold [14], [27]. Further, the mean WTP and its 99% percentile-based confidence interval was determined via the predicted values from the bootstrap interval regression model with 2000 drawings. Additionally, an overall weighted mean was calculated by assuming zero WTP for those who did not respond or who were not willing to pay at all. All above mentioned analyses were carried out with SPSS Release 19.0.0.2 for Windows (SPSS Inc, Chicago, IL, USA) and Stata 11 (StataCorp LP, College Station, TX, USA).

**Table 1:** **Characteristics of participants**

	Missing	WTP Yes	WTP No
	Values	***n*** = 710	***n*** = 741
**Parental characteristics**			
Age (mother), m (sd)	440	38.4 (5.2)	38.9 (4.7)
Age (father), m (sd)	507	41.5 (5.8)	41.8 (5.6)
Maternal overweight, n (%)	130	230 (35.5)	183 (27.2)**
Paternal overweight, n (%)	257	363 (61.2)	370 (61.8)
Maternal obesity, n (%)	130	91 (14.1)	51 (7.6)***
Paternal obesity, n (%)	257	87 (14.7)	78 (13.0)
Maternal WHtR ≥ 0.05, n (%)	706	195 (53.0)	174 (45.2)
Paternal WHtR ≥ 0.05, n (%)	777	228 (67.5)	250 (73.5)
Considering overweight and obesity as a problem, n (%)	3	705 (99.3)	713 (96.6)***
Importance of being thin for being attractive (at least one parent), n (%)	22	432 (61.4)	382 (52.7)**
Consider child too corpulent (at least one parent), n (%)	6	92 (13,0)	47 (6,4)***
High level of maternal health awareness, n (%)	23	407 (58.7)	431 (59.4)
High level of paternal health awareness, n (%)	179	264 (42.0)	273 (42.5)
Smoking (mother), n (%)	29	146 (21.0)	135 (18.5)
Smoking (father), n (%)	158	188 (29.9)	183 (27.6)
Tertiary family education level, n (%)	36	243 (35.2)	225 (31.1)
Monthly household income	199		***
< 2 250€, n (%)		140 (22.1)	200 (32.4)
2 250€ -<4 000€, n (%)		333 (52.5)	287 (46.4)
≥ 4 000€, n (%)		161 (25.4)	131 (21.2)
Single parent, n (%)	15	85 (12.1)	85 (11.6)
**Child characteristics**			
Intervention participant, n (%)	0	399 (56.2)	381 (51.7)*
Age, m (sd)	0	8.06 (0.64)	08.04 (0.63)
Boys, n (%)	0	348 (51.3)	380 (49.0)
Migration background, n (%)	128	209 (32.3)	163 (24.1)**
Overweight, n (%)	30	89 (12.8)	56 (7.7)**
Obesity, n (%)	30	34 (4.9)	21 (2.9)*
Central Obesity, n (%)	29	81 (11.7)	47 (6.5)**
m (mean), sd (standard deviation)			

***<0.001, **< 0.01, *< 0.05.

### Missing data

As a common problem of observational studies, missing data may have an impact on the results. To examine differences between participants with missing outcome variables (general WTP, amount of WTP) and those with complete data, the Mann—Whitney-*U* test for continuous data or the exact Fisher test for categorical data were used. The same applies for differences between records excluded due to missing explanatory variables and records used in the final logistic models.

## Results

From 1 534 parents, 97.8% considered overweight and obesity a serious public health problem with no differences between parents of overweight / obese children and the others. Characteristics of the participants according to their general WTP are displayed in Table 1. 91.1% of the participating parents answered the question concerning their general WTP, 48.8% of them were generally willing to pay. In comparison to the others, those with a general WTP were more frequently overweight and obese themselves, considered overweight and obesity as a problem, found it important to be thin in order to be attractive, considered their child too corpulent and had a higher family income. Children of parents who were generally willing to pay were more frequently in the intervention group of the outcome evaluation, were more frequently overweight, obese and centrally obese and more often had a migration background.

### Willingness to Pay

The stepwise logistic regression analysis for the general WTP resulted in a model including, central obesity of the child, maternal overweight and obesity, migration background of the child and household income. Table 2 shows the adjusted odds ratios for each variable, representing the underlying model.

**Table 2:** **Adjusted odds ratios (OR) for the general willingness to pay**

n = 1 052	OR^1^	***p***-value	95% CI
Central obesity (child)	1.87	0.011	1.16 - 3.03
Maternal obesity	2.36	< 0.001	1.53 - 3.63
Migration background	1.65	0.001	1.24 - 2.19
Monthly household income			
< 2 250€	Reference		
2 250€ -<4 000€	2.09	< 0.001	1.52 - 2.88
≥4 000€	2.63	< 0.001	1.81 - 3.83

CI (confidence limits).

^1^Adjusted for the listed variables in this table.

**Figure 2:**
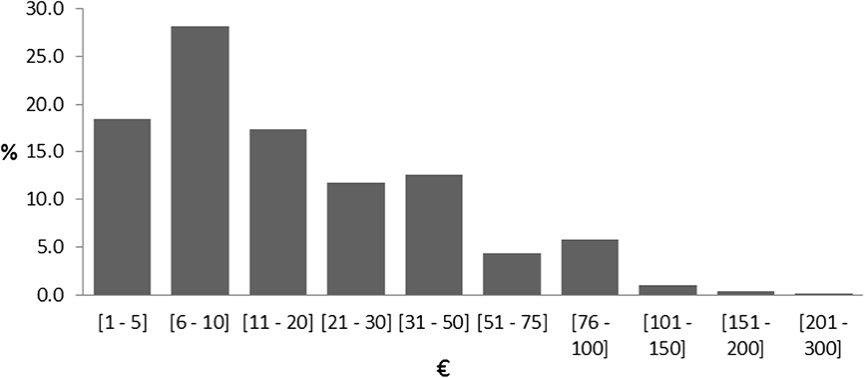
**Categories of the parental willingness to pay (WTP).** Bar chart showing the 10 categories of WTP and the respective percentages referring to n = 710 participants.

Figure 2 visualises the categories of the WTP. It shows a bimodal distribution skewed to the right with the median category at €11 - 20 and two peaks at the categories €6 - €10 and €76 - €100.

The resulting model for the amount of willingness to pay consists of central obesity, migration background and household income. Detailed information of the interval regression model is displayed in Table 3.

**Table 3:** **Bootstrap interval regression model of the amount of willingness to pay**

n = 536	β	***p***-Value	95% CI
Constant	16.13	< 0.001	9.90 - 22.37
Central obesity (child)	15.94	0.002	5.81 - 26.08
Migration background	5.68	0.038	0.31 - 11.05
Monthly household income			
< 2 250€	Reference		
2 250€ -<4 000€	2.52	0.452	-4.05 - 9.08
≥ 4 000€	7.24	0.037	0.46 - 14.37
CI (confidence limits)			

The adjusted mean WTP was €23.04 (99% CI [22.45; 23.75]). The regression coefficients indicate that the average WTP for families who have no child with central obesity, without migration background and with the lowest income category is €16.13. Having a child with central obesity increases the average WTP by €15.94, having a migration background increases average WTP by €5.68 and belonging to the highest income group increases the WTP by €7.42.

710 (44.6%) out of 1 593 participants have stated the amount of their WTP with a mean value of €23.04. Assuming a WTP of zero for those who did not respond and those who were not willing to pay would result in an overall average WTP of €10.27.

### Missing data

Participants with the missing outcome variables, general WTP or amount of WTP (20.7%) differed significantly from the others in terms of being younger, their children had, more often, a migration background and were more frequently overweight, obese and centrally obese.

Those participants with missing explanatory variables for the regression analyses (27.5%) had less frequently a tertiary family education level and had lower household incomes. They were more frequently single parents, mothers were younger and their children were more frequently overweight, obese and centrally obese. There were no statistically significant differences in general WTP or amount of WTP between those with missing explanatory variables and those without.

## Discussion

Preventive measures against childhood overweight and obesity are one of the most important steps towards combating the obesity epidemic [28]. In Germany as well as in the average of all OECD (Organisation for Economic Co-operation and Development) countries, more than half of the population is overweight and obese; posing an immense economic burden on health care systems and national economies. Costs will rise even more in coming years as obesity related diseases set in [29]. Against this background, economic facts concerning prevention have to be collected. Additionally the opinion of the general public has to be made clear to convince governments to act in a more determined fashion.

Information about the cost-effectiveness of preventive measures in general, and in terms of childhood obesity especially, is not easily obtained. Those evaluations which have already been published and are not based on modelling can be counted on two hands [30]. Unfortunately, the outcome measures differ a lot, which makes it difficult to compare the cost-effectiveness of the individual interventions. Waters et al. found no study to include in their Cochrane review in 2011 that contains a formal economic evaluation, but they reminded about the need for cost-effectiveness “to enable well informed decisions about which interventions warrant population-wide implementation” [31].

Though the incremental cost-effectiveness ratio and the individual costs of the URMEL-ICE intervention were published already in 2011 [13], our current study offers a threshold for cost-effectiveness with regard to the prevention of the development of overweight and obesity in children. It could be figured out that the URMEL-ICE intervention, which costs €23.08 per child and year, and cut down the incidence of central obesity by half, is far below the threshold of the average WTP of €23.04 per month (€276.48 per year) which we found in the present study for those who were willing to pay. This remains true, even when considering the overall average WTP of €10.27 per month (€123.24 per year), assuming a WTP of zero for those who were not willing to pay and those who did not respond. The median category of the WTP (€11-20) supports this finding as at least half of the parents were willing to pay an amount of money between the average of those who were willing to pay and the overall average WTP.

### Correlates of willingness to Pay

The parents in this study reveal a high level of awareness of obesity as a major public health problem. Recognition of their own weight problems, especially by mothers, may influence the general WTP. No association was found with the education level, but with income. A higher income was associated with general willingness, as well as with the amount of payment.

Surprisingly, migrants were also generally, and considering the amount, more willing to pay than other citizens. The reasons for that are unclear but perhaps many migrants feel more responsible towards society than non-migrants in Germany do, who take public services for granted. Furthermore, children with a migration background are more likely to be centrally obese (OR 1.68 [1.18; 2.40]), which means 12.1% of the children with migration background are centrally obese versus 7.6% of their peers. This visible sign of too much weight is also associated with the general WTP and the amount of it. Whether this is due to the visibility, or other factors, of central obesity like for instance a higher number of sick days and lower health related quality of life [6] is not clear.

More than half of the respondents are not willing to pay, although they recognise the obesity problems. This may be, inter alia, due to the German health insurance system, where 99.8% of the population are covered, whereof 88% are covered by statutory insurances and 12% in private insurances (Federal Statistical Office of Germany, 2011 Microcensus). But primary prevention is actually only covered in a small part by health insurance; a law on prevention efforts (“Prevention Strengthening Act”) is subject to ongoing political discussion, but until today has not been implemented by the government. Another reason for their denial is possibly the comparatively large burden of taxes and social charges that in 2011 amounted to 37% of the gross domestic product (GDP) in Germany [32].

According to the KiGGS-study overweight and obesity affect 15.4% of children in the age group of seven to ten years in Germany [33]. The overall percentage of overweight and obese children taking part in this WTP study was 10.2%. Hence the majority of participants are parents of children within a normal weight range. On the contrary, participants who did not respond to the WTP questions were more likely to have overweight, obese or centrally obese children.

To be thin in order to be attractive was not associated with WTP in the regression analysis although one would have expected it was. In bivariate analysis it seemed to be highly significant but this was no longer true after adjustment.

### Strengths and limitations

The present study was conducted in an entire federal state of Germany (Baden-Württemberg) and covers many of the different living conditions of families in rural, industrial and urban environments. The large number of participants and amount of data included, allows the control of various results for several co-variables. A special strength is the precision of the anthropometric data of the children, taken by specially trained staff.

To our knowledge, this is the first exploratory analysis of WTP in this thematic area in Europe. Economic data are certainly crucial for decision making in health care [11], and prevention is somehow a neglected field and less the focus of interest, in comparison to therapy. Therefore we consider it very important to learn more about the attitude of the population, especially of the part of the population that is closest to the concerned children, and as it is evident today, is more concerned themselves than ever before.

Nonetheless, some limitations have to be addressed. Due to non- resolvable logistical requirements, no contingent valuation utilizing telephone interviews with elaborated bidding algorithms, was conducted. This may be considered as a methodological limitation, but the influence of the questionnaire methodology used instead on the result in terms of amount of WTP, whether it may be increasing or decreasing, is not quite clear. According to the recommendation of the NOAA (National Oceanic and Atmospheric Administration) panel, face-to-face or telephone interviews are superior to other methods [34]. This may mainly be due to a high selection bias and low response rates in mail surveys. We experienced a response rate of 87% in the baseline measurements and were again expecting a response rate of over 80% for this study (in fact it was 86%). Furthermore, we considered the probability of a socially desirable response behavior higher in the direct contact between interviewer and respondent than in an anonymous questionnaire. Nonetheless, as a consequence, we assumed that the confidence interval for the mean amount of WTP should be of a greater range (99% instead of 95%).

Another limitation is the restricted representativeness. Since only parents of primary school children were asked, the sample is not representative for the general spectrum of the population. In Germany, 31.6% of the adult population have children (Federal Statistical Office of Germany, 2011 Microcensus), meaning that more than two third of the population were not covered by this study. People without children may not be interested in paying for the prevention of overweight and obesity in childhood. On the other hand, the chosen population for this survey was the most relevant, with the highest interest in the subject. In general, contingent valuation questions can be targeted to several groups, the general population or the users of health programmes or even individuals with a disease [35].

Missing data are very common in epidemiological studies and may imply a form of selection bias and lessen the precision of the results. With regard to the missing data analyses in this study, it is possible that those with missing values in the outcome-variables would be more likely to be willing to pay generally and willing to pay a higher amount according to the specific co-variables detected in the regression analyses. For those with missing explanatory variables, no statistical differences in WTP and amount of WTP to those with complete data are shown.

Additionally, it has to be taken into account that this analysis of the WTP was part of the outcome evaluation of an intervention programme, so participants may be sensitized to the subject matter.

## Conclusion

Parental concern about overweight and obesity is obvious with almost 98% of the parents acknowledging that this is a serious public health problem. Almost half of the parents are generally willing to pay for preventive measures and this willingness to pay may even mirror the parental wish for action on behalf of the society or government. This is a strong signal for decision makers and health care officials to improve their efforts in the field of prevention. Future preventive measures should be designed in a sustainable way, with an early onset and constant attendance during the entire education process. Best practice, of course, would be to integrate health promotion and prevention in the curriculum, not only for school-children but for teacher-training as well.

## Authors'contributions

DK, ACS, JMS and other members of the research group planned and organized the Baden-Württemberg study, and were involved in carrying out the measurements in fall 2011. DK performed the statistical analyses, and RK provided expert advice. JMS is the director of the programme "Komm mit in das gesunde Boot - Grundschule" and principal investigator of the Baden-Württemberg Study. DK and RL drafted the manuscript. ACS, TK, RK, and JMS revised the manuscript drafts.

All authors have read and approved the final version of the manuscript.

## Authors’ original submitted files for images

Below are the links to the authors’ original submitted files for images. Authors’ original file for figure 1 Authors’ original file for figure 2
