# Supplementary figures and images for: Parents' willingness to pay for the prevention of childhood overweight and obesity
Source: Health Econ Rev. 2014 Sep 16;4:20. doi: 10.1186/s13561-014-0020-8 (PMC4883987; doi:10.1186/s13561-014-0020-8)

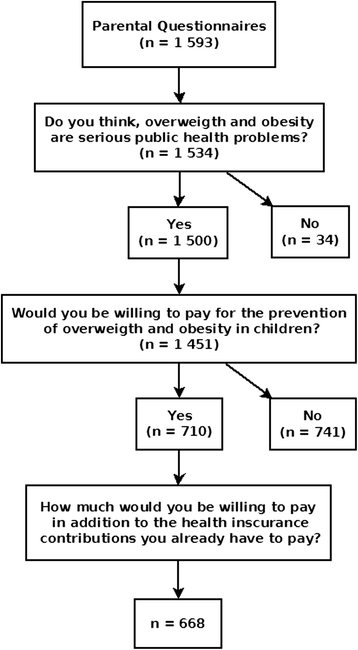

Supplement: Supplementary file 1 — Authors’ original file for figure 1 [file 13561_2014_20_MOESM1_ESM.gif]

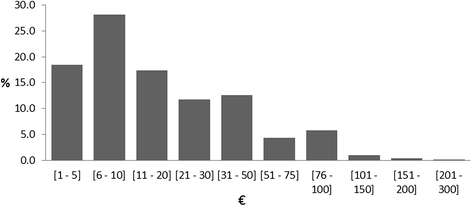

Supplement: Supplementary file 2 — Authors’ original file for figure 2 [file 13561_2014_20_MOESM2_ESM.gif]
